# Brain Responses to Passive Sensory Stimulation Correlate With Intelligence

**DOI:** 10.3389/fnagi.2019.00201

**Published:** 2019-08-14

**Authors:** Anna Horwitz, Marc Klemp, Henrik Horwitz, Mia Dyhr Thomsen, Egill Rostrup, Erik Lykke Mortensen, Merete Osler, Martin Lauritzen, Krisztina Benedek

**Affiliations:** ^1^Department of Drug Design and Pharmacology, University of Copenhagen, Copenhagen, Denmark; ^2^Department of Neuroscience and Pharmacology, University of Copenhagen, Copenhagen, Denmark; ^3^Center for Healthy Aging, University of Copenhagen, Copenhagen, Denmark; ^4^Department of Economics, University of Copenhagen, Copenhagen, Denmark; ^5^Department of Economics, Population Studies and Training Center, Brown University, Providence, RI, United States; ^6^Department of Clinical Pharmacology, Bispebjerg Hospital, University of Copenhagen, Copenhagen, Denmark; ^7^Department of Clinical Neurophysiology, Rigshospitalet Glostrup, Glostrup, Denmark; ^8^Functional Imaging Unit, Department of Clinical Physiology, Nuclear Medicine and PET, Rigshospitalet Glostrup, Glostrup, Denmark; ^9^Department of Public Health, University of Copenhagen, Copenhagen, Denmark; ^10^Research Center for Prevention and Health, Rigshospitalet Glostrup, Glostrup, Denmark

**Keywords:** EEG, gamma power, intelligence, neurocognitive function, steady-state evoked potentials, aging, longitudinal intelligence scores

## Abstract

This study investigates the association between intelligence and brain power responses to a passive audiovisual stimulation. We measure the power of gamma-range steady-state responses (SSRs) as well as intelligence and other aspects of neurocognitive function in 40 healthy males born in 1953. The participants are a part of a Danish birth cohort study and the data therefore include additional information measured earlier in life. Our main power measure is the difference in power between a visual stimulation and a combined audiovisual stimulation. We hypothesize and establish empirically that the power measure is associated with intelligence. In particular, we find a highly significant correlation between the power measure and present intelligence scores. The association is robust to controlling for size-at-birth measures, length of education, speed of processing as well as a range of other potentially confounding factors. Interestingly, we find that intelligence scores measured earlier in life (childhood, youth, late midlife), are also correlated with the present-day power measure, suggesting a deep connection between intelligence and the power measure. Finally, we find that the power measure has a high sensitivity for detection of an intelligence score below the average.

## HIGHLIGHTS

- Intelligence scores are correlated with the change in steady-state evoked power responses from single to double-sensory stimulation (Δ*P*).
- Intelligence scores measured earlier in life (childhood, youth, and late midlife), in addition to scores measured presently in old age, are also associated with present-day Δ*P*.
- The correlations are not driven by observed covariates, including birth size, education, processing speed, or a range of other potentially confounding factors determined after youth, nor other day-specific factors or factors that operate in an additive and constant fashion on the level of steady-state power responses.

## Introduction

The causes of human intelligence is a long-standing scientific topic that can be traced back to Hippocrates, who posited that the study of the mind must begin with the study of the brain. More recently, Spearman (1927) proposed that individual performance in a variety of cognitive activities, including perception, attention, memory, and language is largely determined by a single general ability factor. While psychologists have long been able to measure individual’s level of intelligence by use of cognitive tests, the fundamental driving force of human intelligence is still poorly understood and subject to intense scientific investigation. Furthermore, the development of non-cognitive testing methods of measurement of intelligence is still a largely unexplored terrain.

One prominent theory of intelligence termed the “neural efficiency hypothesis” suggests that brains of more intelligent individuals are more efficient, rather than harder working (Haier et al., 1988, 1992). In particular, the hypothesis provides a possible explanation to the observed phenomenon that, when working on the same cognitive task, higher-IQ individuals elicit a *lower* level of brain activity than lower-IQ individuals.

Several studies using different brain-activity measurement methods have reported negative associations between brain activity and intelligence, supporting the neural efficiency hypothesis (Haier et al., 1992; Anokhin and Vogel, 1996; Jausíovec, 1996, 1998, 2000; Jausíovec and Jausíovec, 2000a, b; Deary et al., 2010). Furthermore, in line with the hypothesis, one study found that differences in brain activation vanish when the difficulty of a given cognitive task is adjusted relative to each test subject’s level of intelligence (Dunst et al., 2014).

However, existing studies investigating the neural efficiency hypothesis have focused on brain activity measured in research subjects as they are actively working on solving cognitively demanding tasks. It therefore still remains an open question if intelligence is also reflected by differences in brain activity measured during a cognitively non-demanding (yet non-resting) stimulation. In particular, it is unknown if the changes in brain activity generated by increasing the number of senses being stimulated with cognitively non-demanding stimuli is associated with intelligence. If so, the evidence supporting the neural efficiency hypothesis may be interpreted in a new light: the differences in brain activity during the solving of cognitively demanding tasks between more and less intelligent individuals may reflect a more fundamental difference in the capacity to process sensory stimuli between the two groups. Furthermore, a reflection of intelligence in brain activity arising from non-demanding stimuli may be used to develop new methods of intelligence assessment that does not involve active task-solving. In light of the difficulties involved in conducting traditional cognitive testing in, e.g., severely cognitively impaired individuals, such methods may 1 day prove to be clinically useful.

Neural activity can be detected by the recording of electrical activity at the scalp using electroencephalography (EEG). Electrical activity of the brain which is measured with EEG in response to a periodically recurring stimulus presented at a fixed rate and which is stable in amplitude and phase is referred to as a “steady-state response” (SSR).

Electroencephalography in general and SSRs in particular are useful tools for investigating the association between physical stimulation, cerebral activity, and cognitive processes (Regan, 1989; Herrmann, 2001; Kappenman and Luck, 2012). They can be interpreted as a natural resonance frequency of the brain, they represent the total effect of entrained background activity and evoked responses, and they reflect primary sensory processing that precede perceptual or attentional processes (Galambos et al., 1981; Karakaş and Başar, 1998; van Deursen et al., 2008). Usefully, SSRs have shown to exhibit a high test–retest reliability (van Deursen et al., 2008). Relevant for the present context, SSRs have been shown to reflect cognitive impairment, such as Alzheimer’s disease, pointing to the existence of an association between SSRs and cognitive function. For example, event studies on SSRs show enhanced auditory SSRs in Alzheimer’s patients with mild to moderate cognitive deterioration (Osipova et al., 2006; van Deursen et al., 2008).

In this study, we propose, and empirically investigate, the hypothesis that passive brain activity – as measured by the power of SSRs in response to a cognitively non-demanding yet non-resting stimulation – increase more in lower-intelligence individuals as a function of the number of senses being stimulated. We thereby propose that the investigation the neural efficiency hypothesis can be extended from a task-solving to a passive context.

Earlier studies investigating the general relationship between intelligence and EEG measures have mainly focused on resting-state lower-frequency brain activity (Anokhin and Vogel, 1996; Jausíovec, 1996; Anokhin et al., 1999; Doppelmayr et al., 2002; Thatcher et al., 2005, 2016); see also the review in Klimesch (1999). More recent studies, however, have turned attention toward the relationship between intelligence and higher-frequency activity (Pahor and Jausíovec, 2014). For example, high-frequency activity has been shown to be especially pronounced during higher cognitive processing in task-based studies. In this study, we focus on high-frequency (i.e., gamma-band) evoked brain activity. There are several reasons for focusing on gamma-band activity (see also Horwitz et al., 2017a). Cognitive function is related to brain activity at various frequencies (see, e.g., Hanslmayr et al., 2008; Roux and Uhlhaas, 2014). Since gamma-band activity is related to age (Duffy et al., 1984; Macpherson et al., 2009, 2012; Muthukumaraswamy et al., 2010; Stranahan et al., 2011; Gaetz et al., 2012; Jessen et al., 2015), and since our cohort consisted of elderly men, gamma band activity appeared to us as a promising candidate frequency band for investigation. Furthermore, since this study is the first to investigate the association between the individual single-to-double sensory power difference and intelligence, we prioritized the collection of as much data as possible for a given frequency band (and thereby increasing the probability of detecting an association for a given frequency), instead of the collection of data for wider set of frequency bands with less data for each frequency. The focus on measurements in the gamma range enabled us to measure a substantial amount data for a relatively large number of participants. Moreover, studies of patients with cognitive decline indicate that gamma-range SSRs may reveal information about cognitive function and decline (see e.g., Osipova et al., 2006; Chee et al., 2009; Hansen et al., 2014). In addition, some evidence suggests that gamma power may be associated with the energy metabolism of the brain (Lachaux et al., 2007). This provides another reason for the use of gamma band activity, since the neural efficiency hypothesis was formulated on the basis of evidence of metabolic rates. Yet, how metabolic changes translates to changes in electrophysiological activity is still unknown. Furthermore, since it is unclear how evoked and task-based brain activity measures are related in the present context, we do not at disregard the possibility that our results can be generalized to other frequency-bands as well.

It should be noted that gamma activity is not unique to a specific cognitive process (Herrmann et al., 2004), but it is associated with a variety of cognitive activities including: perceptual binding (Gray et al., 1989; Singer and Gray, 1995; Pulvermuller et al., 1997; Engel et al., 2001), object recognition (Tallon-Baudry et al., 1998; Keil et al., 1999; Basar et al., 2000; Pesaran et al., 2002), attention (Tiitinen et al., 1993; Gregoriou et al., 2009), arousal (Struber et al., 2000), linguistic processing (Pantev, 1995; Pulvermuller et al., 1995; Eulitz et al., 1996), associative learning (Miltner et al., 1999), consciousness, and REM sleep (Keller et al., 1990; Llinas and Ribary, 1993).

Since brain activity may be affected by a subject’s psychological states (e.g., tiredness, alertness, etc.) or physiological characteristics (e.g., scalp thickness, brain volume, etc.), we focus on the *difference* in a subject’s SSR that occur when we increase the number of senses being stimulated. This allows us to automatically account for possible confounding factors that affect the levels of the SSRs in a constant and additive fashion. Moreover, using a unique feature of our data, we examined the association of our EEG measure with both present and *past* intelligence scores (going as far back as the individual’s childhood). Furthermore, we control for a number of potentially confounding factors that are designed to capture the individual’s general cognitive and physiological state.

The main reason to focus on differences between single-sensory and double-sensory stimulation is to account for omitted variables: the single-sensory stimulation provides information on the “baseline” power response level for each individual. Since a much larger share of the brain is devoted to the visual system than to the auditory system, we expect that the single-sensory visual stimulation will provide the most informative baseline power response measurement. Consequently, we expect that the power responses based on the visual baseline could present a stronger correlation with intelligence than that of the auditory system.

## Materials and Methods

### Empirical Strategy

The main empirical objective of the study is to examine the hypothesis that intelligence is reflected by the relationship between resting-state power responses and the number of senses being stimulated. According to the hypothesis, we expect to find a smaller increase in the power-response in more intelligent individuals. We assess both whether the visual power-difference measure is related to the intelligence measure and whether the auditive power-difference measure is related to the intelligence measure.

Due to the novelty of exploring the use of cognitively non-demanding yet non-passive SSRs to predict cognitive performance, part of the aim of the present study is to develop a statistically sound empirical strategy for this purpose. This included testing the robustness of our findings to investigate if they are driven by our statistical modeling choices.

We attempt to comprehensively tackle the ubiquitous empirical obstacle of confounding factors. In particular, confounding variables may impact both the observed level of intelligence as well as brain measurements and thereby, if not accounted for, obscure the relationship between intelligence and brain measurements. For example, psychological states (tiredness, alertness, etc.) or physiological characteristics (scalp thickness, brain volume, etc.) may be correlated with both day-specific intelligence test-taking ability and EEG responses. We deal with this issue using four strategies, the latter two of which, in our view, represent methodological innovations.

1. We follow the conventional procedure of controlling for a wide range of observed covariates.
2. We focus on a homogenous sample of individuals. All subjects were male and born in 1953 in the same geographical area.
3. We examine not only present-day intelligence measures but also *past* intelligence measures obtained earlier in each individual’s life (going as far back as the individual’s childhood). These past measures of intelligence could not have been affected by many psychological or physiological factors that might have affected the present-day EEG measurement (such as EEG measurement day-specific factors), thus excluding the contribution of these factors to a correlation between intelligence and the EEG measure.
4. We define our brain activity measure in terms of the *change* in the steady-state power response as a function of the number of senses being stimulated, thereby allowing us to automatically account for all factors affecting SSRs in a constant and additive fashion [see also Horwitz et al. (2017a, b) for related strategies].

Each of these strategies are explained in more detail in the following.

#### Control Variables

The level of cognitive abilities is associated with a range of elements, including demographic factors (Stern et al., 1994, 1995; Avlund et al., 2014; Mortensen et al., 2014; Lund et al., 2015), health related factors, and genetic factors, as well as the normal aging process. We therefore account statistically for a wide range of control variables, including birth measures, biomarkers, and the level of education.

#### Sample

As will be explained in section “Participants”, all individuals were male and born in 1953 in the same geographical location. This means that gender, age, and location specific confounding effects are implicitly accounted for. While this reduces the external validity of our analysis, meaning that further research on other demographic groups is needed in order to establish the generalizability of the present findings, it should also increase the internal validity of the study and enable us to provide more precise estimates.

#### Past Intelligence Measures

By investigating if the electrophysiological measurements are associated not only with intelligence at the time of investigation, but also intelligence measured earlier in life, the study assesses whether the association between the EEG measurements and intelligence scores represents a direct association or if it is spuriously associated due to confounding factors such as measurement-day-specific factors or general post-youth lifestyle factors that might have affected both electrophysiological phenomena and intelligence.

#### Explanatory Variable

Our main explanatory variable is the difference in the gamma power response from the single-sensory stimulation to the double-sensory stimulation as a predictive variable for cognitive function. Focusing on the difference, rather than on the levels, of the power of the SSRs to either stimulation serves two main purposes. First, it serves to account for an individual’s baseline power response level, which may be correlated with confounding factors that may be correlated with cognitive function, such as e.g., skull morphology or other physiological parameters. Second, it serves to shed light on the separate question of how brain responses differ between single-sensory and multi-sensory stimulation. The idea of using a differenced measure is also used in earlier work (Horwitz et al., 2017a, b).

### Participants

The study sample comprise of 40 men who were selected from the Metropolit Cohort which contains data on males born in Copenhagen in 1953 (Osler et al., 2006), aged 61–62 at the time of the data collection for the present study (see also Horwitz et al., 2017a). Therefore, the sample represents a homogenous sample (with respect to age, gender, and birth location) of elderly subjects.

This group of people was also examined in Horwitz et al. (2017a) and represent a sub-sample of participants included in the analysis in Horwitz et al. (2017b). Furthermore, the present measurements were taken together with those used in Horwitz et al. (2017a). Therefore, some of the sample description and measurement description sections in this paper (in particular parts of this section and sections “Data Measurement,” “Data Preparation and Analyses,” “Artifacts,” and “Cognitive Assessment”) draws on those of Horwitz et al. (2017a). Further details about the data and related findings can be found in the mentioned articles.

**FIGURE 1:**
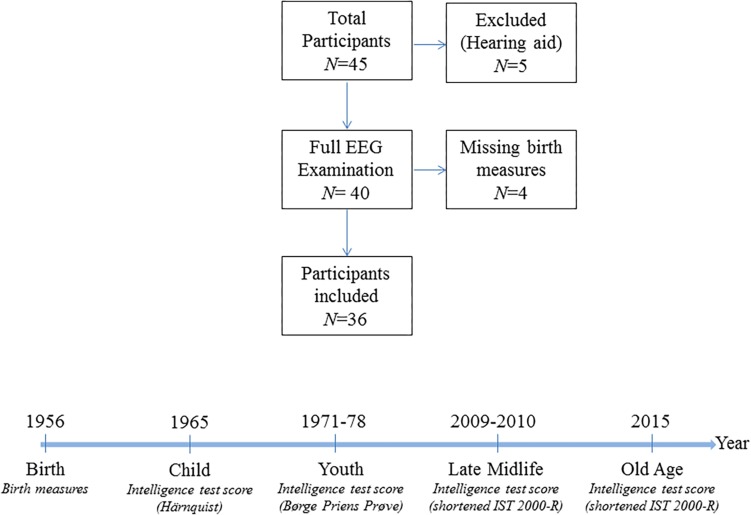
Sample information. The upper panel is a flow chart of the sample selection procedure. The lower panel visualizes the timing of the measurements of the intelligence scores in the cohort and the type of intelligence test used.

All subjects had normal (or corrected-to-normal) vision as well as normal hearing. 84% of the participants were right-handed. The participants were neurocognitively examined with cognitive tests as described in section “Cognitive Assessment” below and they reported no history of psychiatric or neurological disorders. Initially, 45 subjects were investigated, of which five were excluded for this analysis due to missing auditory stimulation caused by the use of hearing aid, resulting in a sample size of 40 individuals. The data does not contain information on birth measures for four participants, and regressions that account for birth measures therefore have a sample size of 36 observations. The sample selection is visualized in Figure 1.

The mean age of the 36 men was 62.02 years, with a standard deviation of 0.37 years. 11% of the subjects were living alone (see Supplementary Table A1). The participants had on average completed 13.4 years of education, and 67% have a long or medium-long education. 67% of the subjects were still employed at the time of study. 97% of the subjects consume alcohol, and 47% smoke (average number of packet years: 14.3). The mean BMI of the participants was 26.3 (SD: 3.3; range: 21.6–35.7). The basic characteristics of the individuals in the sample and the cognitive tests are reported in Supplementary Table A1. The participants’ average MMSE score was 29.6 (SD: 0.6; range: 28–30) and their average Addenbrookes Cognitive Examination (ACE) score was 94.3 (SD: 3.9; range: 82–100), as seen in Supplementary Table A1.

For each participant, the entirety of the present-day measurements was typically conducted within a day (see Horwitz et al., 2017a). The participants met at Rigshospitalet–Glostrup in Denmark at 8 a.m. and left around 4 p.m. The measurements occurred in the following order. First, a blood sample was drawn from the individual. Second, the participant underwent cognitive testing with the following tests and in this order: mini-mental state examination (MMSE), Addenbrooke’s cognitive examination, Trail Making A& B, Symbol-Digit Modalities Test (SDMT), 15 Word Pair, Cambridge Neuropsychological Test Automated Battery (Motor Screening Task, Spatial Recognition Memory, Pattern Recognition Memory, Stockings of Cambridge, Paired Associates Learning, Reaction Time, Rapid Visual Processing), 15 word pair recall, and a shortened version of the Intelligenz-Struktur-Test 2000-R. Third, the participant answered questionnaires on fatigue, sleepiness and depression. Fourth, the participants were given lunch and had a break. Fifth, the participants were measured with EEG and functional magnetic resonance imaging (the order of this changed with every other participant). The only deviations from completing all measurements within a day happened whenever the functional magnetic resonance imaging could not be performed on the day for practical reasons. When this happened, the subject was invited a second time to complete this measurement.

### Stimulation Procedure

Steady-state responses are a sinusoidal response at the (e.g., “on/off”) frequency of a simple visual, auditory or somatosensory stimulus. We used three different stimulation designs. The two first were single-sensory stimulations of either the visual or the auditory system. The third design consisted of a double-sensory simultaneous stimulation of both the auditory and the visual system. In all stimulations, the presentation order to the subjects was randomized. Importantly, to distinguish the brain responses related to the visual and auditory stimulations, visual stimulations were always presented with a flicker-rate of 36 Hz, while auditory stimulations were always presented with a modulation frequency of 40 Hz.

#### Single-Sensory Visual Stimulation

The single-sensory visual stimulation consisted of showing subjects’ a flickering complex illusionary image (i.e., a black-and-white “Rubin’s vase”), which can be perceived either as two opposing faces or as a vase. Rubin’s vase is a special kind of optic illusion, known as an “ambiguous illusion,” and a standard image to exemplify the “figure-ground” distinction made by the brain during visual perception (Rubin, 1915).

**FIGURE 2:**
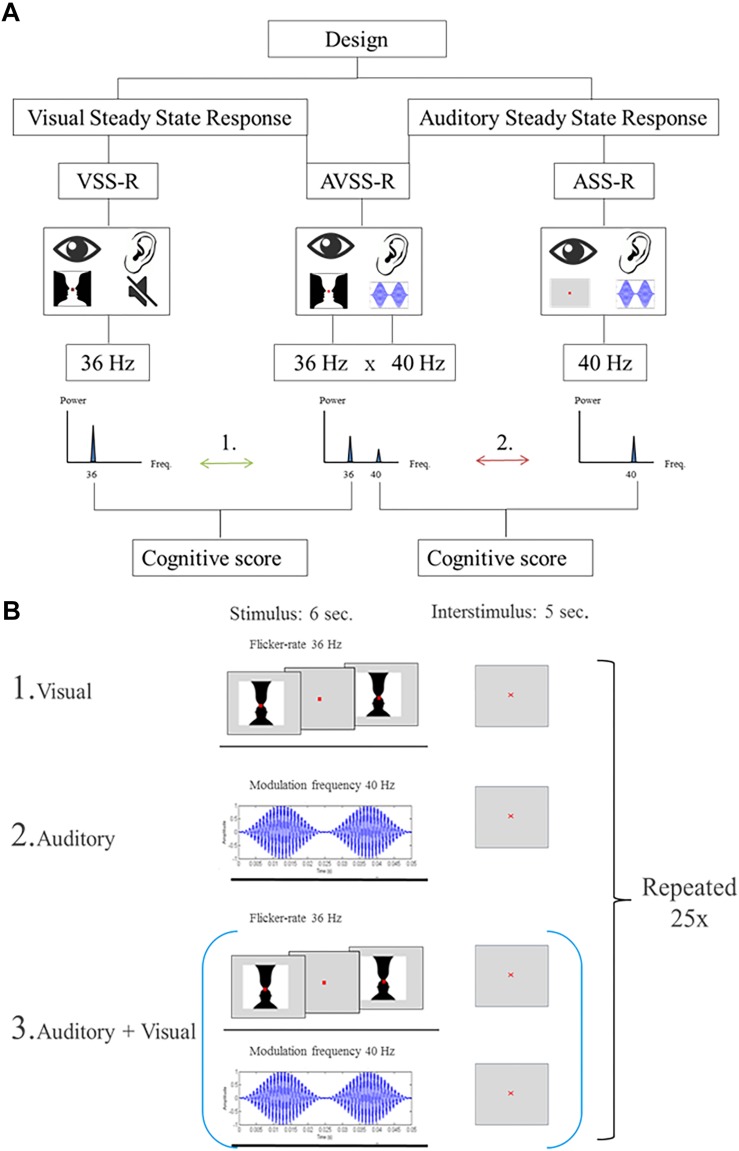
**(A)** Flowchart of the stimulations. The numbers (1, 2) indicate the two main hypotheses, investigated in the paper: Arrow 1: The correlations between 36 Hz-steady state power response to visual, or the audiovisual, stimulation and intelligence. Arrow 2: The correlations between intelligence and 40 Hz-steady state power response to an auditory or audiovisual stimulation. **(B)** Illustration of the stimulation procedure for extracting 6 s stimulation epochs. The start of each stimulation is indicated by a trigger signal. **(B1)** Schematic of the stimulation procedure with an image flickering at a of 36 Hz. **(B2)** Schematic of the stimulation procedure with AM-modulated carrier wave at 1 kHz with a modulation frequency of 40 Hz. **(B3)** Schematic of the combined stimulation procedure with both single-sensory stimulations.

The size of the image was designed to span the central visual area and measured 5 degrees horizontally and 3.25 degrees vertically (8.72 cm × 5.72 cm). Each stimulus consisted of an “on/off design” where the image was interchanged with a gray background with a flicker rate of 36 Hz. Duration of a block was 6 s followed by a 5 s inter-stimulus interval. During the stimulation, a red fixation cross was present (size: 0.33 mm). This block of stimulation and inter-stimulation intervals was repeated 25 times (Figure 2, B1).

#### Single-Sensory Auditory Stimulation

The single-sensory auditory stimulation consisted of exposing participants to an amplitude-modulated (AM) carrier wave at 1 kHz with a modulation frequency of 40 Hz for 6 s. After each 6-s stimulation there was a 5 s inter-stimulation interval and this block of stimulation and interstimuli was repeated 25 times (Figure 2, B2).

#### Double-Sensory Audiovisual Stimulation

The double-sensory stimulation design consisted of presenting the participants to both single-sensory stimulations simultaneously (Figure 2, B3). This consisted of exposing participants to the auditory stimuli mentioned above (carrier wave 1 Hz, frequency modulation 40 Hz), together with the visual “on/off” Rubin’s vase image with a flicker rate of 36 Hz. The duration of a block was 6-s of stimulation which was followed by a 5 s inter-stimulation interval. Each block of stimulation and inter-stimulation was repeated 25 times.

**FIGURE 3:**
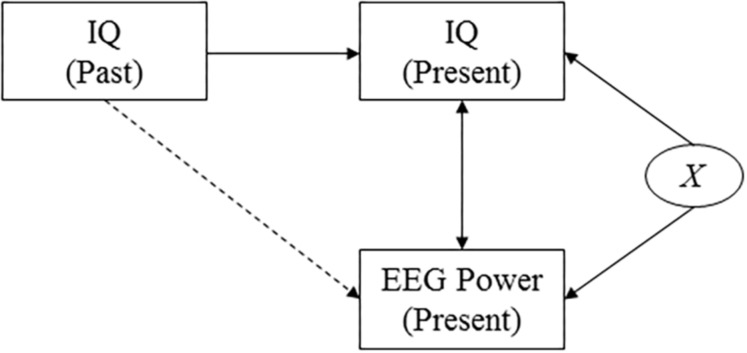
Causal diagram. The diagram shows the assumed causal relationships between cognitive performance at two points in time, EEG power, and a confounding variable. This diagram highlights the effect of an unobserved confounding variable, *X*, that affects both presently measured intelligence scores and presently measured EEG power (for example measurement-day related factors such as subject tiredness). It reveals that by investigating the correlation between past intelligence and presently measured EEG power, the effect of the confounding variable is accounted for.

In line with existing evidence linking general intelligence to frontal lobe function (see, e.g., Duncan, 2005, and references therein), and consistent with the parieto-frontal integration theory of intelligence arguing that the frontal and parietal areas of the cortex are most highly related to intelligence (Jung and Haier, 2007), we mainly focus on frontal-region measurements. However, we include measurements from all scalp areas in our initial analysis.

### Data Measurement

As explained in Horwitz et al. (2017a), we used a soft elastic cap with 64 surface Ag/AgCl electrodes (*Quick-Cap*, *Compumedics*), placed the electrodes according to the international 10–20 system, and connected the cap to a bioamplifier (*SynAmps*, *Compumedics*). We used *Neuroscan* (*Curry7.4 NeuroScan*, *Compumedics*) for signals recording and data processing. The ground electrode is incorporated over the midline frontal region of the cap and the reference was placed between the Cz and Pz-electrodes by the manufacturer. In the data analysis, the signals were re-referenced to bimastoid electrodes (M1, M2).

We kept electrode impedance below 5 kΩ. Furthermore, we registered horizontal and vertical electro-oculographic (EOG) data with two bipolar channels. Moreover, we placed two EKG-electrodes in the heart axis on the chest and two electrodes in submental positions to record EMG-artifacts. We sampled the EEG measurements at 2 kHz with an analog, antialiasing RC low-pass filter at 800 Hz and digitally high-pass and low-pass bandpass filtered the EEG-data offline with a Hann function filter at 0.5 and 250 Hz with a tapering window at 10%. We did not apply any notch filters.

### Data Preparation and Analyses

The data were prepared in the following way. We extracted the event related potential (ERP) averages from epochs of −500 to 6000 ms relative to the stimulus onset and performed a baseline correction using the 500-ms interval before the stimulus onset. Each epoch was extracted at exactly one of the trigger events where a single image stimulus provoked phase-locked averaging (Figure 2). We computed the frequency spectra (in the range from 0.5to 250 Hz) based on the Fourier transformation for ERP averages at all 64 electrodes using CURRY 7.4 Neuroscan.

We computed spectral power values in intervals covering the stimulation flicker rate frequencies used (36 and 40 Hz). The spectral values were multiplied by their frequency to correct for the inverse frequency (1/*f*) characteristic of the typical frequency spectrum. All references to “power” or “log power” in this paper refer to the corrected power, or the natural logarithm of the corrected power.

The visual power values (corresponding to the 36 Hz visual stimulation) were measured in the 35–37 Hz interval and the auditory power values (corresponding to the 40 Hz auditory stimulation) were measured in the 39–41 Hz interval. The visual and auditory power measures are defined as the maximal observed power within each of these non-overlapping intervals, averaged over all electrodes (see the “Definitions” section below). Since the power distribution is very narrow (i.e., not flat) and the maximal power is observed very close to the frequency of the stimulation (36 or 40 Hz), the width of the band should have no consequence for most reasonable ranges of possible bandwidths.

### Artifacts

Initially, the data were visually inspected for obvious anomalies (such as an absence of a signal), which was not found in any of the measurements. As also explained in Horwitz et al. (2017a), the data was corrected offline for artifacts, eye movements, and EKG artifacts using the covariance method in the software CURRY 7.4 Neuroscan. Intervals affected by eye-related movement artifacts were identified using thresholds established using the vertical eye electrodes (VEO), with the lower and upper thresholds set at−200 to 200 μV, respectively. Voltages outside of this interval were considered indicative of eye-related movements, and measurements in all channels in the time range −200 to 500 ms were considered potentially affected by artifacts. In a similar manner, we defined EKG-related artifact-affected intervals in the time range −200 to 500 ms, surrounding a QRS complex detection. The covariance method to correct data in artifact-affected intervals that is implemented in CURRY 7.4 Neuroscan is based on covariance analysis between the artifact channel and each EEG channel, wherein linear transmission coefficients are computed, and, based on these a proportion of the voltage is subtracted from each data point in the artifact interval.

### Cognitive Assessment

The global neurocognitive function was initially assessed with the MMSE and ACE to exclude possible signs of dementia (see Supplementary Table A1). Furthermore, the subjects had their intelligence assessed with a shortened version of the Intelligence Structure Test 2000-R and speed of processing with the Trail-Making Test and the SDMT.

**FIGURE 4:**
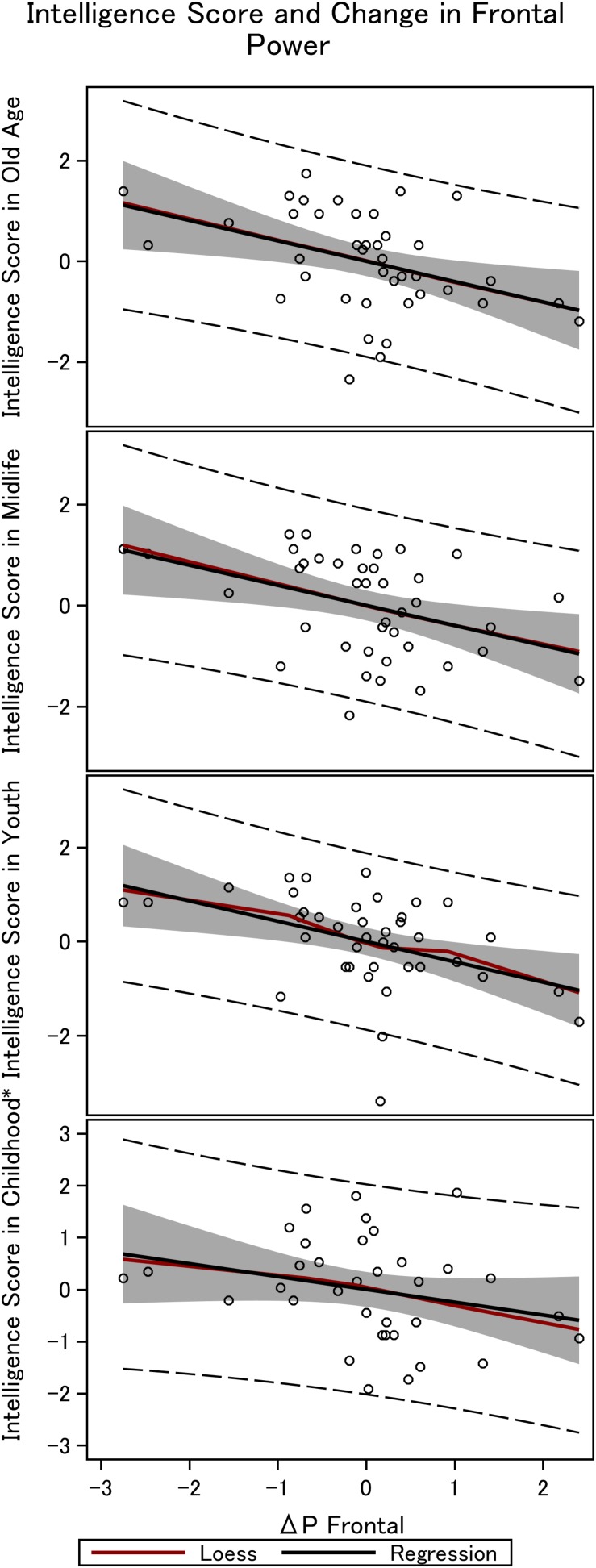
Frontal steady state gamma power and intelligence in the 36 men. The figure depicts the linear fit between intelligence obtained in childhood (∼12 years), youth (∼20 years), midlife (∼56 years), and elderly age (∼62 years), and the difference in power between mono and double-sensory stimulation when investigating the visual response. ^*^Smaller sample size (*N* = 31).

The participants of the Metropolit Cohort (Osler et al., 2006, 2013) have been examined in various ways since birth and consequently we have data of birth measures and cognitive status in childhood, youth as well late midlife, in addition to the present-day intelligence measure (in old age). As described in Horwitz et al. (2017a), the cognitive function was assessed at 12 years of age (1965) with the Härnquist test (Härnqvist, 1968a, b; Osler et al., 2007). The intelligence assessment at 20 years of age (in 1971–78) was a part of the draft board assessment program, assessing the suitability for military service, using the Børge Priens Prøve (BPP) test (Teasdale, 2009; Teasdale et al., 2011). The participants were furthermore cognitively assessed at around 56 years of age, in 2009–10, as part of the Copenhagen Aging and Midlife Biobank project (CAMB) (Osler et al., 2013) with the shortened Intelligenz-Struktur-Test 2000-R (Osler et al., 2013; Mortensen et al., 2014), which comprises three subtests involving verbal analogies, number series, and sentence completion. It should be noted that the intelligence score is not normalized to a population mean of 100. It is defined as the total number of points scored on the Intelligenz Struktur Test (shortened version).

The BPP test consisted of the following categories: letter matrices, verbal analogies, number series and geometric figures. The score is defined as the total number of correct answers out of 78 questions. Scores obtained from this test have been shown to be well correlated with scores from the Wechsler Adult Intelligence Scale (Grønkjær et al., 2019).

The Härnqvist test consisted of the following sub-tests. In a spatial test, the student was asked to pick one out of four three-dimensional figures, which corresponded to a flat, two-dimensional template. In an arithmetic test, the student had to work out the logical order of series of six numbers. In a verbal test, the student had to identify a word out of four alternatives that were antonyms for a given word. Each sub-test included 40 problems.

The Trail Making Test measures participants’ visual attention and ability to switch tasks. It can provide information about visual search speed, scanning, speed of processing, and mental flexibility. For ease of comparison, we multiplied the Trail Making Test scores with−1, implying that a higher score on this (“negated”) scale reflects higher cognitive function. The SDMT test measures participant attention, concentration, and speed of information processing (Giovagnoli et al., 1996; Sheridan et al., 2006). By including this measure as a control variable, we aim to account for possible cognitive impairments.

As shown in Table 1, the measures of intelligence were significantly correlated over individual’s life times, indicating that the different measures capture common fundamental characteristics of individual’s intelligence levels. Meanwhile the correlations between the cognitive tests are not perfect, suggesting that the various tests potentially measure different aspects of intelligence at different points in life and that the intelligence tests are affected by measurement noise such as day-specific factors.

**TABLE 1:** The results of linear regression models of the intelligence score obtained in childhood (∼12 years), youth (∼20 years), late midlife (∼56 years), and at current examination (∼62 years), and the difference between single-sensory power and double-sensory power (Δ*P*_*V,F*_) when investigating the visual system.

	**Intelligence score in**							
	**Old age (2015)**		**Late midlife (2000)**		**Youth (1971–1978)**		**Child (1965)**	
	**shortened IST 2000-R**		**shortened IST 2000-R**		**Børge Prien**		**Härnquist**	
	**1**	**2**	**3**	**4**	**5**	**6**	**7**	**8**
Δ*P*_*V,F*_	−0.41^∗∗∗^ (0.09)	−0.50^∗∗∗^ (0.09)	−0.40^∗∗∗^ (0.10)	−0.52^∗∗∗^ (0.09)	−0.43^∗∗∗^ (0.09)	−0.51^∗∗∗^ (0.09)	−0.25^*^ (0.10)	−0.28^*^ (0.10)
BMI at birth		−0.27^*^ (0.13)		−0.30^*^ (0.11)		−0.13 (0.16)		−0.07 (0.19)
Semi-partial, Δ*P*_*V,F*_	0.16	0.24	0.16	0.26	0.19	0.25	0.07	0.08
Adjusted *R*^2^	0.14	0.21	0.14	0.23	0.16	0.20	0.04	0.02
Number of individuals	40	36	40	36	40	36	35	31

Each column represents one linear regression model. Columns 2, 4, 6, and 8 are adjusted for birth measures. The robustness tables when adjusting for single-sensory power level in the occipital lobe and years of education is shown in the appendix, using a stepwise regression design, establishing that the correlation between power and intelligence scores are robust to accounting for these factors as well. The linear fits are depicted in Figure 4. The semi-partial *R*2 statistics for the main explanatory variables are shown together with the adjusted *R*2 statistics. Robust standard errors are shown in parentheses. ^*^p < 0.05, ^∗∗∗^*p* < 0.001. All specifications include a constant which is omitted from the table.

### Related Data

Information on education, categorized by years of education, as well as birth weight and length are also available and used as control variables in the analysis.

### Statistics

To estimate the relationship between cognitive function and the power measure we employ a series of multiple linear regression models. These models enable us to estimate partial associations between our outcome variable (i.e., the intelligence test score) a number of explanatory variables (including the power measure).

We assess statistical significance with two-sided *t*-tests and use a significance level of 5%. We calculate confidence limits for proportions with Wald’s method. Standard errors in all regression models are based on the Eicker-Huber-White estimator. Since *t*-statistics, and thus *p*-values, are depend only on the coefficient estimates and standard errors, we report only coefficient estimates and standard errors in the tables. We also report the adjusted *R*2 values, as well as the semi-partial *R*2 values for the main explanatory variables. All statistical analyses are performed in *SAS 9.4*.

The linear regression models used to investigate the relationship between cognitive function and SSRs takes the following form:

$$y_{i}={\text{β}}_{0}+{\text{β}}_{1}\,\left(x_{2,i}-x_{1,i}\right)\,+{\text{β}}_{2}\,x_{1,i}+{𝑪}_{3,i}^{\prime }\,{𝛃}_{𝟐}+{\text{ε}}_{i},$$

where *y*_*i*_ is a measure of cognitive function (i.e., an intelligence test score), *x*_1,i_ is the average power response in the frontal region in response to single-sensory stimulation, i.e., based on the power response to the 36 Hz flicker rate when stimulating the vision (corresponding to *P*_*S,F,V*_ defined below), and *x*_2__,_*_*i*_* is the analogous measure in response to double-sensory stimulation, i.e., based on the power response to the 36 Hz flicker rate (corresponding to *P_*D*__,__*F,V*_* defined below). The term ***C***_3_ is a vector of control variables, including a measure of global cognition and measures of processing speed. We also include the level, *x*_1_, alongside the difference, *x*_2_ –* x*_1_, which is our main variable of interest (corresponding to Δ*P*_*V,F*_ defined below), to account for the fact that the difference may be correlated with the level, which may in turn be independently correlated with cognitive function. To assess the robustness of the results, we specify the model with varying subsets of the explanatory variables.

Earlier studies have shown that birth measures correlate with cognitive ability (Sorensen et al., 1997; Breeman et al., 2015). Likewise, education may affect intelligence scores. Furthermore, both birth factors and education may potentially affect SSRs, causing them to be potential confounding variables. We therefore investigate the association when controlling for birth BMI (or birth weight and length separately) and years of education in the models. Furthermore, we analyze the robustness of the observed association between intelligence scores and SSRs by investigating the correlation between cognitive functions measured earlier in life, at around 12 years, 20 years and at 56 years of age, and SSRs, while also assessing the robustness of those correlations to controlling for birth measures and education.

Measured intelligence can thus potentially be affected by a range of factors that could also affect the measured power response, such as the health or mood of the subject on the day of the examination. To verify that our findings do not only capture correlations that are due to such confounding factors but really reflect a deeper biological mechanism, we investigate the correlation between power and intelligence measurements performed in the past (i.e., in the subject’s youth, and midlife). A causal diagram that presents the hypothesized underlying causal structure of the variables is shown in Figure 3. The diagram implies that finding correlations between, on the one hand, cognitive performance as measured earlier in life and, on the other hand, presently measured EEG data, rule out the potential confounding effects of a wide range of factors that are determined later in life, including day-specific factors.

Furthermore, we control for biological heterogeneities between subjects that are captured by differences in birth weight and length. Finally, since education may affect intelligence, we control for the number of years of education. The possible effect of education on measured intelligence is highlighted in Table 2 which shows that the correlation between intelligence scores measures and education stronger at later stages in life.

**TABLE 2:** The results of Pearson correlation coefficients between the intelligence test scores obtained in childhood, youth, late midlife, and in elderly age (i.e., the present, and years of education).

	**Intelligence score in**			
	**Child (1965) Härnquist**	**Youth (1971-78) Børge Prien**	**Late midlife (2000) shortened IST 2000-R**	**Old age (2015) shortened IST 2000-R**
Years of Education	0.21 (*N* = 35)	0.47^∗∗^ (*N* = 40)	0.50^∗∗∗^ (*N* = 40)	0.55^∗∗∗^ (*N* = 40)
Intelligence score in child (1965) Härnquist	–	0.55^∗∗∗^ (*N* = 35)	0.71^∗∗∗^ (*N* = 35)	0.70^∗∗∗^ (*N* = 35)
Intelligence score in youth (1971–1978) Børge Prien		–	0.64^∗∗∗^ (*N* = 40)	0.65^∗∗∗^ (*N* = 40)
Intelligence score in late midlife (2000) IST 2000-R			–	0.89^∗∗∗^ (*N* = 40)

*The table shows that the correlation between intelligence measures and education are stronger at later stages in life. ^∗∗^*p* < 0.01, ^∗∗∗^*p* < 0.001.*

All variables were standardized, meaning that, for each variable, we subtracted the mean and divided with the standard deviation.

### Definitions

The power-measure used is frequency-corrected, i.e.,

P=M⁢f,

where *P* is corrected power, *M* is measured power – both measured in squared microvolts, i.e., (μ*V*)2 – and *f* is the corresponding frequency. We introduce a second subscript to identify the electrode when relevant. The main analysis focuses on the frontal brain area power response accounting for the set of electrodes F2, F4, F6, F8, F1, F3, F5, F7, FZ, FP1, FPZ, FP2, FC5, FC3, FC1, FCZ, FC2, FC4, and FC6 (shortened “*F*”). We furthermore refer to the set of electrodes Oz, O1, O2 as the occipital area (shortened “*O*”), the set of electrodes PZ, PO4, PO6, PO8, P2, P4, P6, P8, PO3, PO5, PO7, P1, P3, P5, and P7 as the parietal area (shortened “*P*”), and the set of electrodes T7, T8, TP7, TP8, FT7, and FT8 as the temporal area (shortened “*T*”).

We now introduce a number of subscripts that indicate the stimulation type, the brain area over which the power measure is averaged, and the frequency at which the power is measured:

1. The first subscript indicates if the measurements come from the single-sensory (labeled “S”) or the double-sensory (labeled “D”) stimulation.
2. The second subscript indicates the observed frequency spectrum and is either labeled “*V*” for the spectrum around 36 Hz, which is relevant for the visual stimulus, or “*A*” for the spectrum around 40 Hz, which is relevant for the auditory stimulus.
3. The third subscript indicates the brain area, using the labels “*F*,” “*O*,” “*P*,” and “*T*” from above.

Thus, for example, the variable *P_*S*__,__*V*__,F_* denotes the average power for the frontal region measured at 36Hz, obtained with the single-sensory stimulation. Finally, we introduce the differenced measures, which are defined as,

$$\Delta \,P_{i,j}=P_{D,i,j}-\,P_{S,i,j},$$

for a frequency spectrum, *i*, and a brain area *j*. Thus, for example, the variable Δ*P_*V*__,F_* denotes the single-to-double-sensory difference in average frontal-region power observed in the 36Hz frequency spectrum for the visual stimulus.

Figure A1 illustrates an example of the steady-state EEG data output for one participant. Data represented in the time domain, as a butterfly plot, is shown in Panel A (filtered at 0.5–250 Hz) and panel B (filtered at 30–70 Hz, i.e., the gamma range). The spatial distribution of the SSR with a clear peak at 36 Hz in Panel C. The time frequency spectrogram for the frontal electrodes is shown in panel D. Furthermore, spatial distribution in the central electrode (*F*_*z*_) is shown to demonstrate the existence of two peaks: one at 36 Hz and another at 40 Hz in the double-sensory stimulation, corresponding to the stimulation frequencies (Panel E). Finally, the position plot of the spatial distribution shown in the range of 30–45 Hz is illustrated in Panel F. Overall, the example illustrates that the stimulation procedure generates clear peaks at frequencies corresponding to those of the stimulation.

As mentioned in section “Related Data”, we calculated each subjects’ individual change in gamma power from single-sensory to double-sensory stimulation (i.e., Δ*P*_*V,j*_) for each of four scalp areas as well as for the total scalp.

### Ethics Statement

This study was approved by the local ethical committee (Danish: *De Videnskabsetiske Komiteer for Region Hovedstaden*) and registered by the Danish Data Protection Agency. All participants provided written informed consent.

## Results

### Intelligence Score and SSRs for the Total Scalp and Four Scalp Regions

We first conduct an analysis of intelligence and the SSRs obtained across all electrodes on the scalp (Supplementary Table A2). We find that the difference in the power for the total brain in response to the visual stimulation (i.e., Δ*P*_*V,Total*_) is borderline negatively correlated with intelligence (*R*2: 0.07; *p* = 0.054) and that the difference in the power for the total brain in response to the auditory stimulation (i.e., Δ*P*_*A,Total*_) is insignificantly correlated with intelligence. When sub-dividing the scalp into four main regions of interest (i.e., the frontal, parietal, temporal and occipital scalp regions) we find that – in line with existing evidence linking general intelligence to frontal lobe function and in accordance with the parieto-frontal integration theory of intelligence – the measure for the frontal brain is the one that is most strongly related to intelligence (Supplementary Table A2, column 2). If one considers each of the tests of the coefficients of interest in columns 1–5 as separate families of hypotheses and perform a conservative Bonferroni correction (multiplying *p*-values with 5), Δ*P*_*V,F*_ (with *p* < 0.0001) remains statistically significant.

Furthermore, in a “horse race” regression with all four regions of interest included in the same regression, the coefficient on the difference in frontal power is negative and the only statistically significantly coefficient out of the measures for any of the scalp regions (Supplementary Table A2, column 6–7). When investigating the correlation between intelligence and the difference between the power difference in the frontal and the power difference of the rest of the brain (i.e., Δ*P_*V*,__*Total*_* − Δ*P*_*V,F*_), we find that Δ*P*_*V,F*_ is significantly negatively correlated and that the difference for the remaining electrodes was insignificant (Supplementary Table A2, column 8). Reassuringly, the coefficient remains significant when correcting for multiple hypothesis testing with the conservative Bonferroni correction.

These preliminary results suggest that, in accordance with our hypothesis, power differences are correlated with intelligence. Furthermore, they corroborate existing evidence linking general intelligence to frontal lobe function and are consistent with the parieto-frontal integration theory of intelligence, establishing that the association is pronounced for the frontal scalp region. In the main part of the analysis, we therefore continue to focus on the frontal brain region.

### Intelligence and Visual Evoked Frontal Gamma Power

In this section, all EEG measurements relate to the visual evoked response, that is, the frontal scalp area at 36 Hz. As already explained, we focus on the difference between the single-sensory and the double-sensory frontal power (Δ*P*_*V,F*_, i.e., *P*_*S*__,_*_*V,F*_* subtracted from *P_*D*__,V__,F_*).

#### Intelligence and the Difference in Single-Sensory and Double-Sensory Power

We find a negative association between the difference in frontal power between double-sensory and single-sensory visual stimulation (Δ*P_*V*__,F_*) and intelligence in childhood, youth, late midlife and at present time (in old age). Figure 4 depicts the raw correlation. Furthermore, Table 1 presents a series of regression models for the intelligence scores at different life stages while alternating between including and excluding the birth BMI control variable. Column 1 of Table 1 establishes that Δ*P_*V*__,F_* is significantly negatively correlated with the present intelligence score on the shortened IST 2000-R. The parameter estimate of −0.41 means that an increase in Δ*P_*V*__,F_* of 1 SD is associated with 0.41 fewer points on the standardized shortened IST 2000-R score (semi-partial *R*2: 0.16; *p* < 0.0001). This association is robust to controlling for birth BMI (column 2). Moreover, the same patterns of robust significant correlations are observed for intelligence earlier in life, as measured by the shortened IST 2000-R test in late midlife (Table 1, columns 3–4) and by the BPP in youth in year 1973 (Table 1, column 5–6) as well by the Härnquist-test in childhood in year 1965 (column 7–8).

Supplementary Table A3 shows the results of regressions including the single-sensory level of the four main region of interest as well as the power differences for the occipital area and the total scalp area. In all specifications, the coefficient on our main explanatory variable remains significantly negative. Furthermore, the other measures are insignificant.

#### Intelligence Test Sub-Components

The finding of significant correlations between Δ*P_*V*__,F_* and intelligence raises the question whether some dimensions of intelligence are more strongly correlated with Δ*P_*V*__,F_* than others. Table 3 investigates this question by splitting the present shortened IST 2000-R score into three subcomponents, namely the “sentences,” the “analogies” and the “numbers” part of the test.

**TABLE 3:** The results of linear regression models of the test scores from each of the tree subcomponents of the shortened IST 2000-R test and the difference between single-sensory power and double-sensory power (Δ*P*_*V,F*_).

**Shortened IST 2000-R**	**Sentence completion score**		**Analogy score**		**Numeric score**	
	**1**	**2**	**3**	**4**	**5**	**6**
Δ*P*_*V,F*_	−0.32^∗∗^ (0.10)	−0.42^∗∗∗^ (0.10)	−0.35^∗∗^ (0.10)	−0.46^∗∗∗^ (0.09)	−0.35^∗∗^ (0.13)	−0.41^∗∗^ (0.14)
BMI at birth		−0.33^*^ (0.16)		−0.40^∗∗^ (0.12)		−0.07 (0.13)
Semi-partial *R2* for Δ*P*_*V,F*_	0.10	0.17	0.12	0.23	0.12	0.15
Adjusted *R*2	0.08	0.15	0.10	0.24	0.10	0.10
Number of individuals	40	36	40	36	40	36

Each column represents one linear regression model. Column 2, 4 and 6 adjust for birth measures. Robust standard errors in parentheses. ^*^p < 0.05, ^∗∗^*p* < 0.01, ^∗∗∗^*p* < 0.001. All specifications include a constant which is omitted from the table.

Table 3 establishes that Δ*P_*V*__,F_* is robustly significantly correlated with the test scores in all the three sub-components of the test, namely the sentences part (*R*2 = 0.10; *p* = 0.003), the analogies part (*R*2 = 0.12; *p* = 0.001), and the numbers part (*R*2 = 0.12; *p* = 0.005).

#### Additional Control Variables

Furthermore, we also control for biomarkers with possibly confounding effects on intelligence (see Supplementary Table A5). The coefficient of interest stays significant in all the specifications, indicating that the association between the EEG measurements and intelligence represents a non-superficial relationship. In particular, since both presently measured intelligence scores and EEG measurements can potentially be correlated with a range of biological factors, such as homocysteine, vitamin-D, or cholesterol, we control for measurements of these factors in addition to a range of other potential confounding variables.

When including the measured blood markers as control variables, we find that the main explanatory variable (Δ*P_*V*__,__*F*_*) remains significantly correlated with intelligence (Supplementary Table A5). Interestingly, the levels of B_12_-vitamin, homocysteine and total cholesterol are significantly negatively correlated with intelligence and even so when controlling for birth measures (Supplementary Table A5). Global cognition is known to be impacted by dietary circumstances and thereby plausibly vitamin levels. Even when controlling for global cognition measured with ACE, the coefficient on Δ*P_*V*__,F_* remain significantly correlated with intelligence (column 7, *R*2 = 0.15, *p* = 0.02). In addition, since hemoglobin, folate, methylmelonate, creatinine, P-glucose (HbA1c), P-glucose, HDL, LDL, and TG may confound the correlation, we also control for these factors. Overall, our estimates show that Δ*P_*V*__,F_* is significantly and robustly correlated with intelligence, and that this correlation is not driven by a wide range of potentially confounding factors.

#### Robustness to Exclusion of Possible Outliers, Alternative Samples, and Alternative Specifications

The scatterplots depicting the raw associations between our explanatory variable and intelligence may suggest that a few observations play a large role for the precision of the estimation. We therefore investigated the robustness of the findings with regards to eliminating the six observations for which Δ*P_*V,F*_* > −1 or Δ*P_*V,F*_* < 1 (results are available upon request). Reassuringly, we find that the results are robust to the exclusion of these observations. In particular, we re-estimated the specifications in Table 1 for the restricted sample and found that, in all specifications, the coefficient of interest remains statistically significantly negative [the only exception being the specifications underlying column 1 in which the coefficient is very close to significance (*p* = 0.051)].

Furthermore, Supplementary Table A4 in the Supplementary Materials establishes that the main findings are robust to excluding one individual with zero correct answers in the numerical sub-test of the shortened IST 2000-R test, restricting the sample to the 36 individuals with known birth measures while not including the birth measures in the regression, including the birth BMI components (birth weight and length) as separate variables, controlling for years of education, and controlling for the single-sensory occipital visual power.

### Accounting for Speed of Processing

In Table 4, we include measures of processing speed as control variables. We find that our power measure remains significantly correlated with intelligence and that the model explains even more of the variation in intelligence scores, accounting for the larger number of explanatory variables (see the adjusted *R*2). This is especially true for Δ*P_*V*__,F_* when controlling for the Trail Making A and B test scores (semi-partial *R*2 = 0.29), as well as the SDMT test score (semi-partial *R*2 = 0.19). This establishes that the difference between single-sensory and double-sensory stimulation (Δ*P_*V*__,F_*) captures a correlation with intelligence that is not depending on the subjects’ capabilities of attention, concentration, or their speed of information processing, leaving, presumably, variation in the intelligence test score that mainly reflect crystalized intelligence.

**TABLE 4:** The table shows the results of linear regression models of the test scores of intelligence 2015 (present ∼62 years) and difference between the single and double-sensory power (Δ*P*_*V,F*_) for the visual evoked response when controlling for each of the four cognitive test and measures of speed of processing.

	**Intelligence score (shortened IST 2000-R) in old age (2015)**					
	**1**	**2**	**3**	**4**	**5**	**6**
Δ*P*_*V,F*_	−0.44^∗∗∗^ (0.11)	−0.47^∗∗∗^ (0.11)	−0.40^∗∗^ (0.14)	−0.42^∗∗^ (0.12)	−0.48^∗∗∗^ (0.11)	−0.38^∗∗^ (0.11)
BMI at birth	−0.23# (0.11)	−0.22^*^ (0.10)	−0.17 (0.12)	−0.17 (0.13)	−0.21# (0.12)	
Global cognition, ACE	0.46^∗∗∗^ (0.10)				0.33^∗∗^ (0.10)	0.29^∗∗^ (0.10)
Negated Trail-Making A Score^a^		−0.04 (0.19)			0.10 (0.17)	0.16 (0.19)
Negated Trail-Making B Score^a^		0.57^∗∗^ (0.19)			0.46^*^ (0.19)	0.31# (0.17)
Symbol-Digit Modalities Test Score			0.38^*^ (0.16)		−0.12 (0.21)	−0.01 (0.18)
Working verbal memory				−0.45# (0.24)	−0.51^∗∗^ (0.16)	−0.56^∗∗^ (0.18)
Long-term verbal recall				−0.01 (0.23)	0.54^*^ (0.26)	0.47^*^ (0.22)
Semi-partial *R2* for Δ*P*_*V,F*_	0.25	0.29	0.19	0.21	0.32	0.24
Adjusted *R*2	0.40	0.47	0.34	0.37	0.55	0.53
Number of individuals	36	36	36	36	36	40

Each column represents one linear regression model. ^*a*^Measured by test completion-time. Robust standard errors in parentheses. #*p* < 0.1, ^*^p < 0.05, ^∗∗^*p* < 0.01, ^∗∗∗^*p* < 0.001. All specifications include a constant which is omitted from the table.

Supplementary Table A6 establishes that Δ*P*_*V,F*_ is not significantly related to other aspects of cognition (however, “long-term verbal recall” is significantly related in a linear regression model but it does not present a significant Pearson correlation coefficient).

### Auditory Evoked Frontal Gamma Power and Intelligence

In this section, all EEG measurements are related to the auditory response at 40 Hz, i.e., the difference between *P_*S*__,A__,F_* and *P_*D*__,A__,F_* (i.e., Δ*P_*A*__,F_*). In this analysis, we also correct for the hearing threshold of each individual.

We do not find any associations between the difference for the total brain (Δ*P_*A,T*__*otal*_*) and intelligence (see Supplementary Table A7). None of the three regions of interest corresponding to the default network are independently significantly correlated and have a low semi-partial *R*2.

Supplementary Table A7 column 5–6 shows the correlation when controlling for both the differences in the visual and the auditory steady state power response. It shows that Δ*P_*V*__,F_* remains robustly negatively correlated with intelligence, even when controlling for the single-sensory level (*R*2 = 0.15, *p* < 0.0001). Furthermore, Δ*P_*A*__,F_* is also negatively, but insignificantly, associated with intelligence.

The results of linear regression models of the intelligence score obtained in childhood (∼12 years), youth (∼20 years), late midlife (∼56 years), and at current examination (∼62 years) on the difference between single-sensory power and double-sensory power (Δ*P*_*A,F*_) for the auditive system are shown in Table 5. The table establishes that when controlling for birth BMI and single-sensory temporal power the coefficient is significant on 5% level.

**TABLE 5:** The results of linear regression models of the intelligence score obtained in childhood (∼12 years), youth (∼20 years), late midlife (∼56 years), and at current examination (∼62 years), and the difference between single-sensory power and double-sensory power (Δ*P*_*A,F*_) when investigating the auditive system.

	**Intelligence score in**											
	**Old age (2015) shortened IST 2000-R**			**Late midlife (2000) shortened IST 2000-R**			**Youth (1971–1978) Børge Prien**			**Child (1965) Härnquist**		
	**1**	**2**	**3**	**4**	**5**	**6**	**7**	**8**	**9**	**10**	**11**	**12**
Δ*P*_*A,F*_	−0.06 (0.15)	−0.11 (0.20)	−0.54^*^ (0.24)	0.09 (0.18)	0.05 (0.25)	−0.67^*^ (0.27)	−0.04 (0.15)	−0.17 (0.22)	−0.69^∗∗^ (0.23)	0.06 (0.17)	0.10 (0.18)	−0.62^*^ (0.27)
BMI at birth		−0.11 (0.14)	−0.07 (0.14)		−0.16 (0.13)	−0.10 (0.12)		0.04 (0.16)	0.09 (0.17)		0.02 (0.20)	0.14 (0.18)
Single-sensory temporal power			−0.44# (0.25)			−0.73^*^ (0.28)			−0.52^*^ (0.21)			−0.76^∗∗^ (0.22)
Semi-partial *R2*; Δ*P*_*A,F*_	0.00	0.01	0.06	0.01	0.00	0.10	0.00	0.02	0.09	0.00	0.01	0.09
Adjusted *R*2	−0.02	−0.03	−0.01	−0.02	−0.04	0.08	−0.02	−0.03	0.01	−0.03	−0.06	0.06
Number of individuals	40	36	36	40	36	36	40	36	36	35	31	31

Each column represents one linear regression model. Column 2–3, 5–6, 8–9, and 11–12 are adjusted for birth measures and single-sensory power level in the temporal lobe in a stepwise fashion. The semi-partial *R*2 for the main explanatory variables are shown together with the models adjusted *R*2. Robust standard errors in parentheses. #*p* < 0.1, ^*^p < 0.05, ^∗∗^*p* < 0.01. All specifications include a constant which is omitted from the table.

Overall, we found that the most statistically significant association between intelligence and frontal processing is found for the visual response. The auditory response variable is not significant in our data except for specifications in which we include the single-sensory auditory power as a separate explanatory variable.

### Lower Intelligence Scores and Differences in Single-Sensory and Double-Sensory Power

**FIGURE 5:**
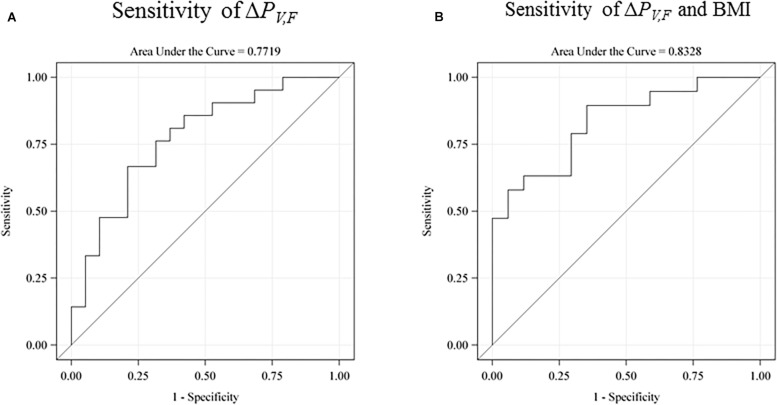
The receiver operating characteristic (ROC) sensitivity curve of the main explanatory variable (the difference between the single-sensory and the double-sensory gamma steady state response) as a predictor of a low intelligence score (defined as below the sample mean). **(A)** The raw sensitivity obtained without use of any control variables. 77% (95% CI: 62–92%). **(B)** The sensitivity when including birth BMI. The overall sensitivity in this case is 0.83% (95% CI: 70–96%). The auditory response is not shown but has a raw sensitivity of 65% (95% CI: 47–83%).

We investigated the sensitivity of the test for prediction of low intelligence scores defined as below the sample mean (see Figure 5). The raw receiver operating characteristic (ROC) sensitivity of Δ*P_*V*__,F_* was 77% (95% CI: 62–92%). When including birth BMI, the joint sensitivity was 0.83% (95% CI: 70–96%).

## Discussion

Our findings demonstrate a significant and robust association between single-sensory-to-double-sensory changes in frontal power responses and cognitive function, indicating that the brain activity reaction to changes in sensory stimulation is related to intelligence. Furthermore, the difference in the frontal steady-state power response from a single-sensory to a double-sensory stimulation can predict low intelligence with 83% sensitivity when paired with information on birth BMI. We also found that the association between our EEG measure and intelligence is not driven by possible outliers, the subject’s years of education, speed of processing, and other factors.

We proposed that the investigation of the neural efficiency hypothesis can be extended from a task-solving to a passive context. We interpret our findings as supportive of our hypothesis and of the neural efficiency hypothesis in general. In particular, our results suggest that passive brain activity – as measured by the power of SSRs in response to a cognitively non-demanding yet non-resting stimulation – increase more in lower-intelligence individuals as a function of the number of senses being stimulated. Our working hypothesis could be relevant for brain activity at any frequency band. It is therefore entirely possible that associations between single-sensory-to-double-sensory differences in power and intelligence can be found for other frequency bands and further studies are needed to investigate this.

Furthermore, our results are in line with existing evidence linking general intelligence to frontal lobe function and they are consistent with the parieto-frontal integration theory of intelligence. In particular, we find that the power-difference measure for the frontal brain area is more strongly related to intelligence than power differences for the total scalp. Furthermore, our findings were consistent with our expectation of a stronger correlation with intelligence for the visual system than for the auditory system. In particular, the explanatory variable for the auditory system has a lower raw sensitivity of 65% and a lower joint sensitivity with birth BMI of 67%.

The study is, to our knowledge, the first to find an association between intelligence test scores and single-to-double-sensory differences in brain activity. The analysis also indicates that the steady-state power-responses to *exclusive* stimulation of the auditory or the visual system do not have nearly as much explanatory power for intelligence as the variables that include data from the combined stimulation procedure. As explained in the introduction, we assumed that the visual response provided the most informative baseline response for the main explanatory variable. However, our hypothesis does predict that even the auditory-to-audiovisual response should be correlated with intelligence in sufficiently large samples. The present study is not able to neither confirm nor refute that possibility. In particular, while the auditory-to-audiovisual measure is correlated with intelligence in the same way as the visual-to-audiovisual measure, it is not statistically significant. Further studies using larger samples may be able to determine whether our prior is correct.

While our stimulation setup is non-demanding (meaning that subjects are not asked to solve a task while being measured) we speculate that the setup may potentially still reflect the brain’s activity changes in response to an increase in cognitive demand. In particular, higher brain effort may be required in order to “filter out” uninformative stimuli (i.e., effectively noise) when two senses are being stimulated rather than one.

Meanwhile, the ability to filter out noise may also occur completely automatically in a way that is not directly related to cognitive function. In this latter scenario, we could expect the present methodology to provide a better fit in patients with Alzheimer’s disease, who have been shown to be characterized by lower cortical inhibitory function. If so, the present methodology may prove useful in the early diagnosis of Alzheimer’s disease and related types of dementia. Our findings may therefore also suggest that existing neurophysiological investigations of Alzheimer’s disease by auditory steady-state stimulation alone could benefit from a double-sensory stimulation procedure (van Deursen et al., 2008).

The study sample is unique in that participants are all male, born in the same year and were born (and therefore, plausibly, for the most part grew up in) the same local area of Denmark. This is an important factor because earlier studies point out that cognitive ability in late midlife is associated with demographic factors which may in turn contribute to individual differences in health and early aging (Gottfredson, 2004; Mortensen et al., 2014; Bolzenius et al., 2015). Other studies have found sex differences and educational differences in cognitive ability across life stages (Mortensen et al., 1989; Mortensen and Gade, 1993; Mortensen and Kleven, 1993), which suggests that social inequality may also contribute to demographic differences in cognitive ability. Given that such factors may also affect the steady state power response and thereby obscure or bias an observed association between intelligence and power, the homogeneity of the present sample helps to account for these factors.

We find significant correlations between education and cognitive function but also that the steady state power response remains significantly correlated with intelligence even when controlling for education. This indicates that the electrophysiological measures capture correlations with intelligence that are at least not entirely driven by variations across subjects in terms of their educational achievements (Figure 3).

Furthermore, since earlier studies have shown that perinatal insult, and birth measures correlates with cognitive ability (Sorensen et al., 1997; Breeman et al., 2015), we control for birth measures, and find consistently robust results. In addition, we control for a range of blood measures that all could affect cognition, again finding robust results. These findings largely exclude the possibility that the correlation between intelligence and SSRs can be explained by confounding factors that are determined after the youth of the subjects, strongly suggesting that the association between the main explanatory variable and intelligence test scores is not spurious.

A unique data feature is the existence of past intelligence test scores. Our EEG measure is not only correlated with present-day intelligence test scores but, reassuringly, also intelligence test scores measured earlier in life, i.e., in childhood, youth and late midlife. These findings indicate that the correlation between the EEG measure and intelligence cannot be attributed to measurement-day specific factors.

Overall, we interpret our findings as supportive of our hypothesis that passive brain activity – as measured by the power of SSRs to a cognitively non-demanding yet non-resting stimulation – increase more in lower-intelligence individuals as a function of the number of senses being stimulated. We argue that our findings support the neural efficiency hypothesis in general. Moreover, our analysis suggests that SSRs may be used in the development of useful supplements to cognitive neuropsychological testing.

## Conclusion

In this study, we propose, and find empirical support for, the hypothesis that passive brain activity – as measured by the power of SSRs in response to a cognitively non-demanding yet non-resting stimulation – increase more in lower-intelligence individuals as a function of the number of senses being stimulated.

The study presents two main findings. First, the study establishes that the power response difference between a single-sensory and a double-sensory stimulation can be a useful indicator of neurocognitive performance. The difference between the visual steady state power in response to the single-sensory (i.e., visual) stimulation and the double-sensory (i.e., audiovisual) stimulation, Δ*P_*V*__,F_*, is significantly negatively correlated with neurocognitive performance in the sample of 61–62 year old healthy men not suffering from dementia. The steady-state power-response difference presents a sensitivity of 83% of detection of a low intelligence score (i.e., a score below the mean of the sample) when paired with information on birth BMI.

Second, the study finds that Δ*P_*V*__,F_* is associated not only with intelligence at the time of investigation, but also intelligence measured earlier in life, at childhood, youth and late midlife and robustly and more significantly so when controlling for demographic and biological factors including years of education, birth measures, and blood measures. Thereby, we conclude with greater confidence that the association between power and intelligence is not spurious and that it indicates a deep biological mechanism.

Overall, the study suggests that the SSR power differences obtained with single-sensory stimulation and double-sensory stimulation is a useful indicator of neurocognitive performance. We conclude that audiovisual SSR measures may prove clinically useful in assessing intelligence and, by extension, cognitive decline.

## Ethics Statement

This study was approved by the local ethical committee (De Videnskabsetiske Komiteer for Region Hovedstaden) and registered by the Danish Data Protection Agency. All participants provided written informed consent.

## Author Contributions

AH contributed to the conception, design of the work, data acquisition, and drafted the work. AH and MK analyzed the data. AH, MK, HH, MT, EM, MO, ER, ML, and KB contributed to the interpretation of data and revising it critically for important intellectual content. AH, MK, HH, MT, EM, MO, RO, ML, and KB approved the final version to be published. AH, MK, HH, EM, MO, ER, ML, and KB prepared the agreement to be accountable for all aspects of the work in ensuring that questions related to the accuracy or integrity of any part of the work are appropriately investigated and resolved.

## Conflict of Interest Statement

The authors declare that the research was conducted in the absence of any commercial or financial relationships that could be construed as a potential conflict of interest.
