# Supplementary material for: Brain Responses to Passive Sensory Stimulation Correlate With Intelligence
Source: Front Aging Neurosci. 2019 Aug 14;11:201. doi: 10.3389/fnagi.2019.00201 (PMC6702683; doi:10.3389/fnagi.2019.00201)
Supplement: Supplementary file 1 [file Data_Sheet_1.pdf]

# Supplementary Material

| | Variable | Mean (SD) | Range |
|---|---|---|---|
| | **Early measures** | | |
| **Birth Parameters** | Birth Weight, kg | 3.5 (0.6) | 2.2 – 4.6 |
| | Birth Length, cm | 52.6 (2.4) | 46.0 – 58.0 |
| | Birth BMI | 12.7 (1.25) | 10.4 – 16.0 |
| **Intelligence Score at 12 Years** | Härnquist-test | 72.4 (17.1) | 42.0 – 104.0 |
| **Intelligence Score at 18-20 Years** | Børge Prien Prøve (BPP) | 44.5 (9.8) | 12.0 – 58.0 |
| **Intelligence Score at 57 Years** | IST -2000-R | 35.3 (10.5) | 13.0 – 50.0 |
| | **Cognitive Today** | | |
| **Global Cognitive Tests** | Mini-Mental State Examination | 29.6 (0.6) | 28.0 – 30.0 |
| | Addenbrooke's Cognitive Examination | 94.3 (3.9) | 82.0 – 100.0 |
| **Speed of Processing and Executive Function** | Trail-Making Test A | 34.8 (10.1) | 22.0 – 61.0 |
| | Trail-Making Test B | 81.0 (25.0) | 46.0 – 148.0 |
| | Symbol-Digit Modalities Test | 44.4 (8.04) | 28.0 – 64.0 |
| **Intelligence Score -Testing** | I-S-T 2000-R (total) | 32.6 (11.1) | 8.0 – 50.0 |
| | I-S-T 2000-R (sentences) | 12.6 (3.2) | 8.0 – 18.0 |
| | I-S-T 2000-R (analogues) | 10.3 (3.6) | 4.0 – 17.0 |
| | I-S-T 2000-R (numbers) | 9.8 (6.2) | 0.0 – 20.0 |
| | **Socioeconomic** | | |
| **Life Style-Related Variables** | BMI, kg/m$^2$ | 26.3 (3.3) | 21.6– 35.7 |
| | Weight, kg | 85.9(12.5) | 67.0 – 118.0 |
| | Height, cm | 180.5 (5.6) | 170.0 – 191.0 |
| | Circumference, cm | 96.0(8.8) | 82.0 – 123.0 |
| | Systolic BP, mm Hg | 141.6 (17.7) | 111.0 – 190.0 |
| | Systolic BP, mm Hg | 86.3 (10.8) | 62.0 – 109.0 |
| | Right handed | 33 [91.7 %] | 0 – 1 |
| **Family** | Married | 32 [89 %] | 0 – 1 |
| | Children | 1.8 (0.9) | 0 – 4 |
| | Siblings | 1.9 (2.6) | 0 – 12 |
| **Education and Work** | Years of education | 13,4 (2.4) | 7.0 – 25.0 |
| | Long higher education | 10 [29 %] | 0 – 1 |
| | Medium-long higher education | 14 [40 %] | 0 – 1 |
| | Vocational | 10 [29 %] | 0 – 1 |
| | Non-vocational | 1 [3 %] | 0 – 1 |
| | Employed | 23 [67 %] | 0 – 1 |
| | Retirement pension | 12 [33 %] | 0 – 1 |
| | Unemployed | 2 [6 %] | 0 – 1 |
| **Alcohol and Tobacco Habits** | Consume alcohol | 35 [97 %] | 0 – 1 |
| | Smoking weekly | 17 [47 %] | 1.0 – 28.0 |
| | Packet-years smoked | 14.3 (11.4) | 0.0 – 115.0 |

Note: Values are means (with SD in parentheses) or counts (with percentages in brackets). Abbreviations: BMI, Body-Mass Index; MMSE, Mini-Mental State Examination.

**Table A1:** Characteristics of the sample and neurocognitive measures ($N$=36).

| | Intelligence Score in Old Age (2015) I-S-T-2000-R | | | | | | | |
|---|---|---|---|---|---|---|---|---|
| | 1 | 2 | 3 | 4 | 5 | 6 | 7 | 8 |
| $\Delta P_{V,Total}$ | -0.26[#] (0.13) | | | | | | | |
| $\Delta P_{V,F}$ | | -0.41*** (0.09) | | | | -0.45*** (0.11) | -0.45*** (0.11) | -0.39*** (0.08) |
| $\Delta P_{V,P}$ | | | -0.13 (0.09) | | | 0.14 (0.33) | 0.17 (0.34) | |
| $\Delta P_{V,T}$ | | | | -0.19# (0.10) | | -0.06 (0.11) | -0.06 (0.11) | |
| $\Delta P_{V,O}$ | | | | | -0.10 (0.14) | -0.02 (0.36) | -0.04 (0.36) | |
| *HT* | | | | | | | -0.00 (0.03) | |
| $\Delta P_{V,(Total\text{-}F)}$ | | | | | | | | 0.09 (0.08) |
| Semi-Partial $R^2$ | 0.07 | 0.16 | 0.02 | 0.04 | 0.01 | 0.14 [a] | 0.15 [a] | 0.15[a] |
| Number of Individuals | 40 | 40 | 40 | 40 | 40 | 40 | 37 | 40 |

[a] The semi-partial $R^2$ is only shown for $\Delta P_{V,F.}$

Robust standard errors in parentheses. $^{\#}p$<0.1,* $p$<0.05, ** $p$<0.01, *** $p$<0.001. All specifications include a constant which is omitted from the table.

**Table A2:** The table shows the results of linear regression models of the test scores of intelligences 2015 (i.e., at ~62 years of age) and the visual evoked power responses for the total brain response as well as the four main regions of interest. Column 2–4 show a "horse race" regression with all four regions of interest included in the same model, the difference in frontal power remains significantly negatively correlated while the coefficients on the differences in the other brain regions are not significant. Furthermore column 8 show that $\Delta P_{V,F}$ is significantly negative correlated when controlling for the power response for the rest of the Brain, $\Delta P_{V,(Total\text{-}F)}$ (i.e., $\Delta P_{V,Total}$ - $\Delta P_{V,F}$).

| Main Explanatory Variable: Frontal Power Difference ($\Delta P_{V,F}$) | Intelligence Score in Old Age (2015) I-S-T-2000-R 1 | 2 | 3 | 4 | 5 | 6 | 7 |
|---|---|---|---|---|---|---|---|
| $\Delta P_{V,F}$ | -0.41*** | -0.38*** | -0.46*** | -0.44*** | -0.40*** | -0.43*** | -0.39*** |
| | (0.09) | (0.09) | (0.09) | (0.09) | (0.09) | (0.10) | (0.08) |
| $P_{M,V,F}$ | | 0.08 | | | | | |
| | | (0.10) | | | | | |
| | | $R^2$: 0.01 | | | | | |
| $P_{M,V,O}$ | | | -0.16* | | | | |
| | | | (0.06) | | | | |
| | | | $R^2$: 0.03 | | | | |
| $P_{M,V,P}$ | | | | -0.13# | | | |
| | | | | (0.06) | | | |
| | | | | $R^2$: 0.02 | | | |
| $P_{M,V,T}$ | | | | | 0.05 | | |
| | | | | | (0.09) | | |
| | | | | | $R^2$: 0.00 | | |
| $\Delta P_{V,O}$ | | | | | | 0.07 | |
| | | | | | | (0.16) | |
| | | | | | | $R^2$: 0.01 | |
| $\Delta P_{V,(Total-F)}$ | | | | | | | 0.09 |
| | | | | | | | (0.08) |
| | | | | | | | $R^2$: 0.01 |
| Semi-Partial $R^2$ for $\Delta P_{V,F}$ | 0.16 | 0.14 | 0.19 | 0.18 | 0.16 | 0.16 | 0.15 |
| Adjusted $R^2$ | 0.14 | 0.13 | 0.14 | 0.13 | 0.12 | 0.12 | 0.13 |
| Number of Individuals | 40 | 40 | 40 | 40 | 40 | 40 | 40 |

Robust standard errors in parentheses. # $p$<0.1,* $p$<0.05, ** $p$<0.01, *** $p$<0.001. All specifications include a constant which is omitted from the table.

**Table A3:** The table shows the results of linear regression models of the test scores of intelligence 2015 (present ~62 years) and the visual evoked power responses for the Frontal Power Difference ($\Delta P_{V,F}$) when controlling for the single-sensory power level in the four main regions of interest. Furthermore column 7 show that $\Delta P_{V,F}$ is significantly negative correlated when controlling for the power response for the rest of the Brain, $\Delta P_{V,(Total-F)}$ (i.e., $\Delta P_{V,Total} - \Delta P_{V,F}$ ).

| | Intelligence Score in Old Age (2015) I-S-T-2000-R | | | | |
|---|---|---|---|---|---|
| | 1 | 2 | 3 | 4 | 5 |
| $\Delta P_{V,F}$ | -0.40*** | -0.43*** | -0.50*** | -0.25* | -0.32** |
| | (0.09) | (0.09) | (0.10) | (0.12) | (0.11) |
| Birth Weight | | | -0.55* | | |
| | | | (0.21) | | |
| Birth Length | | | 0.49* | | |
| | | | (0.23) | | |
| BMI at Birth | | | | -0.13 | -0.15 |
| | | | | (0.13) | (0.13) |
| Years of Education | | | | 0.47** | 0.45** |
| | | | | (0.14) | (0.14) |
| Single-Sensory Occipital Power | | | | | -0.17** |
| | | | | | (0.05) |
| Semi-Partial $R^2$ for $\Delta P_{V,F}$ | 0.17 | 0.19 | 0.25 | 0.06 | 0.09 |
| Adjusted $R^2$ | 0.15 | 0.17 | 0.21 | 0.35 | 0.36 |
| Number of Individuals | 39 | 36 | 36 | 36 | 36 |

Robust standard errors in parentheses. * $p<0.05$, ** $p<0.01$, *** $p<0.001$. All specifications include a constant which is omitted from the table.

**Table A4:** Robustness table. The table establishes that the main findings are robust to excluding one individual with zero correct answers in the numerical sub-test of the IST 2000 R (short) test (column 1), restricting the sample to the 36 individuals with known birth measures while not including the birth measures in the regression (column 2), including the birth BMI components (birth weight and length) as separate variables (column 3), controlling for years of education (column 4), controlling for the single-sensory occipital visual power (column 5).

| | Intelligence Score in Old Age (2015) I-S-T-2000-R | | | | | | | | |
|---|---|---|---|---|---|---|---|---|---|
| | 1 | 2 | 3 | 4 | 5 | 6 | 7 | 8 | 9 |
| $\Delta P_{V,F}$ | -0.46*** (0.11) | -0.52*** (0.12) | -0.50*** (0.10) | -0.52*** (0.11) | -0.51*** (0.10) | -0.51*** (0.09) | -0.35* (0.13) | -0.32* (0.12) | -0.37** (0.12) |
| BMI at Birth | Yes | Yes | Yes | Yes | Yes | Yes | Yes | Yes | No |
| Hemoglobin | Yes | | | | | | Yes | Yes | Yes |
| Vitamin B12, Homocysteine, Pholate, methylmalonic acid, creatinine | | Yes | | | | | Yes | Yes | Yes |
| D-25-OH-Vitamin D | | | Yes | | | | Yes | Yes | Yes |
| P-Glucose (HbA1c) | | | | Yes | | | Yes | Yes | Yes |
| P-Glucose | | | | | Yes | | Yes | Yes | |
| Total Cholesterol, HDL, LDL, TG | | | | | | Yes | Yes | Yes | Yes |
| Global cognition, ACE | | | | | | | | Yes | |
| Semi-Partial $R^2$ for $\Delta P_{V,F}$ | 0.20 | 0.23 | 0.24 | 0.24 | 0.26 | 0.28 | 0.15 | 0.15 | 0.14 |
| Adjusted $R^2$ | 0.22 | 0.18 | 0.20 | 0.19 | 0.21 | 0.26 | 0.28 | 0.39 | 0.19 |
| Number of Individuals | 36 | 36 | 36 | 36 | 36 | 36 | 36 | 36 | 39 |

Robust standard errors in parentheses. $^{\#}$ $p$<0.1,* $p$<0.05, ** $p$<0.01, *** $p$<0.001. All specifications include a constant which is omitted from the table.

**Table A5:** The table shows the results of linear regression models of the test score of presently measured intelligence (i.e., at ~62 years) and the difference between the single and double sensory power when controlling for potentially confounding blood measures. Note that even though controlling for all blood variables, and birth measures at the same time seems excessive in light of the sample size, the coefficient of interest remains statistically significant at the 1% level even in that case.

| | MMSE | Global cognition, ACE | Negated Trail-Making A Score [a] | Negated Trail-Making B Score [a] | Symbol-Digit Modalities Test Score | Working verbal memory | Long-term verbal recall |
|---|---|---|---|---|---|---|---|
| $\Delta P_{V,F}$ | -0.23 ($N$=40) | -0.09 ($N$=40) | -0.14 ($N$=40) | -0.06 ($N$=40) | -0.19 ($N$=40) | -0.15 ($N$=40) | -0.26 ($N$=40) |

**Table A6:** The table shows the Pearson correlation coefficients between our main variable of interest ($\Delta P_{V,F}$) and seven cognitive test scores. MMSE: Mini-mental state examination.

| | Intelligence Score in Old Age (2015) I-S-T-2000-R | | | | | |
|---|---|---|---|---|---|---|
| | 1 | 2 | 3 | 4 | 5 | 7 |
| $\Delta P_{A,Total}$ | 0.04 | | | | | |
| | (0.10) | | | | | |
| $\Delta P_{A,F}$ | | -0.06 | | | -0.21 | -0.02 |
| | | (0.15) | | | (0.27) | (0.27) |
| $\Delta P_{A,T}$ | | | -0.06 | | | |
| | | | (0.15) | | | |
| $\Delta P_{A,P}$ | | | | 0.11# | | |
| | | | | (0.06) | | |
| $P_{M,A,T}$ | | | | | -0.19 | -0.05 |
| | | | | | (0.24) | (0.22) |
| $\Delta P_{V,F}$ | | | | | | -0.40*** |
| | | | | | | (0.10) |
| Semi-Partial $R^2$; $\Delta P_A$ | 0.00 | 0.00 | 0.00 | 0.01 | 0.01 | 0.00 |
| Semi-Partial $R^2$; $\Delta P_{V,F}$ | | | | | | 0.15 |
| Number of Individuals | 40 | 40 | 40 | 40 | 40 | 40 |

Robust standard errors in parentheses. # $p$<0.1,* $p$<0.05, ** $p$<0.01, *** $p$<0.001. All specifications include a constant which is omitted from the table.

**Table A7:** The table shows the results of linear regression models of the test scores of intelligence scores 2015 (present ~62 years) and the auditory evoked power responses for the total brain response as well as the main regions of interest. Column 5 - 6 show shows the correlation when controlling for both the differences in the visual and the auditory steady state power response. It shows that $\Delta P_{V,F}$ remains robustly negatively correlated with intelligence, even when controlling for the single-sensory level.

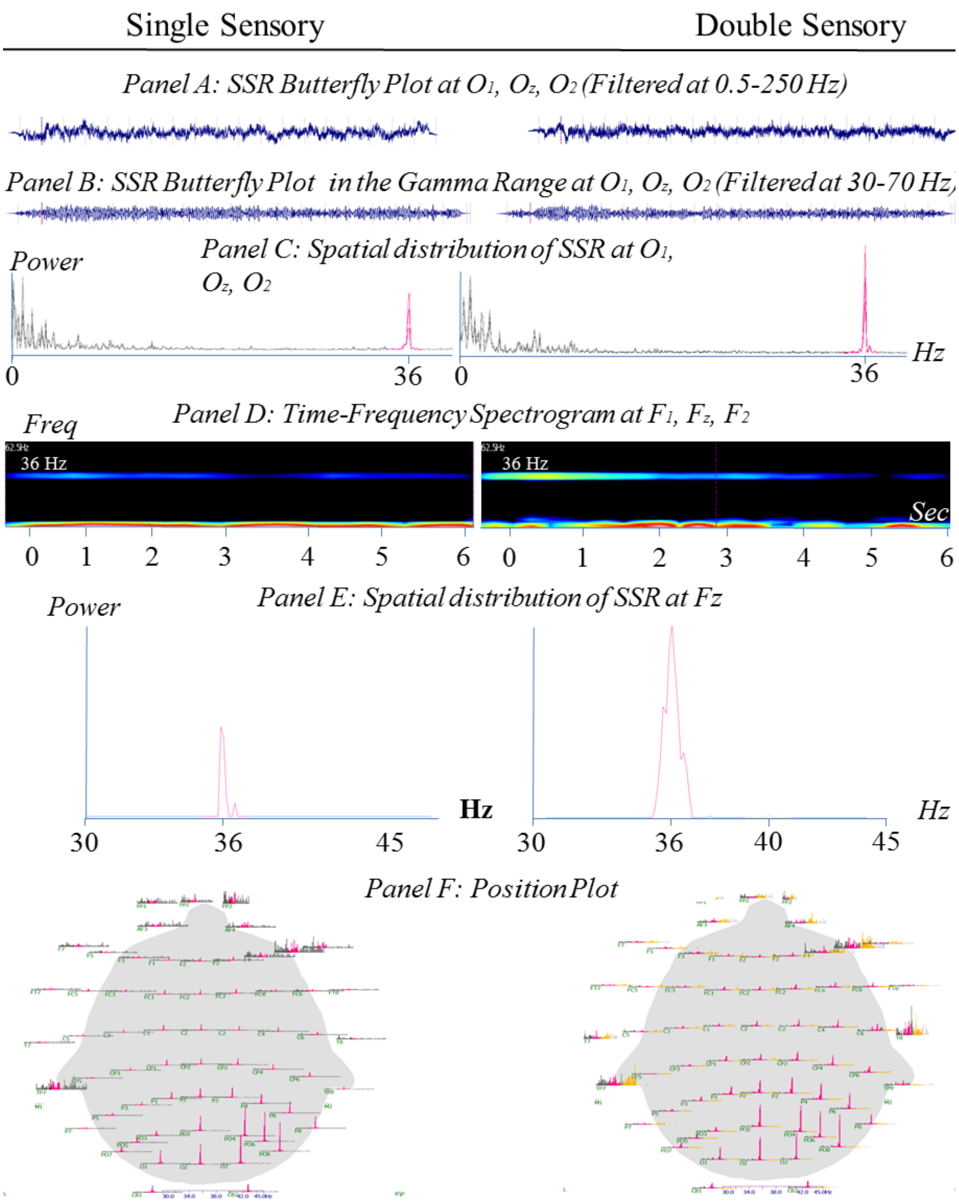


**Figure A1**. Illustration of the electrophysiological response (steady-state evoked potential) in the single-sensory and the double-sensory stimulation with a focus on the visual response. The data is shown for the same subject. ***Panel A:*** the amplitude in the time domain filtered at 0.5–250 Hz. A butterfly plot of the response in $O_1$, $O_2$, $O_z$ with Reference to M1, M2 is shown. ***Panel B:*** the time domain data in the Gamma range when filtered at 30–70 Hz. A butterfly Plot of the response in $O_1$,$O_2$, $O_z$ with Reference to M1, M2 is shown. ***Panel C:*** the distribution of the SSR for the occipital electrodes ($O_1$, $O_2$, $O_z$ ) data filtered at 0.5-250 Hz. A clear peak at 36 Hz is seen for both single-sensory and double-sensory stimulation with an indication of greater response in the double-sensory stimulation. ***Panel D:*** the time frequency spectrogram for the frontal electrodes ($F_1$, $F_2$, $F_z$ ) data filtered at 0.5–250 Hz. Resolution 256 ms and max frequency shown 62.5 Hz. ***Panel E:*** the spatial distribution of the SSR for the central electrode ($F_z$) data filtered at 0.5–250 Hz. Notes that there is zoomed so only data for 30–45 Hz is shown and the scale is 10 times higher. We see a clear peak at 36 Hz at single-sensory stimulation vs to peak at 36 Hz and 40 Hz in the double-sensory stipulation in response to the stimulation frequency. ***Panel F:*** the position plot of the spatial distribution filtered at 0.5–250 Hz and shown in the range of 30–45 Hz.
